# Clinical Impact of Weight Loss During Hospitalization on Prognosis After Pancreatic Surgery

**DOI:** 10.7759/cureus.69427

**Published:** 2024-09-14

**Authors:** Shota Kuwabara, Takumi Nakaya, Keita Ishido, Yuma Aoki, Kazuyuki Yamamoto, Yasuhito Shoji, Akira Fukunaga, Tatsunosuke Ichimura, Hiroto Manase, Satoshi Hirano

**Affiliations:** 1 Surgery, Asahikawa Red Cross Hospital, Asahikawa, JPN; 2 Gastroenterological Surgery II, Hokkaido University Faculty of Medicine, Sapporo, JPN

**Keywords:** nutrition, pancreatic cancer, prognosis, surgery, weight loss

## Abstract

Purpose

This study aimed to elucidate the relationship between early-stage weight loss (WL) during hospitalization after pancreatic surgery and prognosis and investigate risk factors affecting WL.

Methods

We included 68 patients diagnosed with pancreatic ductal adenocarcinoma (PDAC) who underwent radical surgery. The %WL value was calculated based on the percentage of body weight at discharge compared with the body weight at admission. High WL was defined as %WL >10%. We initially evaluated the association between %WL and postoperative survival using the Kaplan-Meier method. Subsequently, we analyzed the factors affecting %WL using a logistic regression model.

Results

In terms of overall survival (OS), the high %WL group exhibited a significantly worse prognosis than the low %WL group (p=0.043). Univariate analysis revealed a hazard ratio of 2.244 (95% confidence interval (CI), 1.006-5.006; p=0.048) for high %WL in relation to overall survival. Multivariate analysis identified an operative time >450 min (odds ratio, 17.8; 95% confidence interval, 1.01-312.42; p=0.049) and postoperative complications (odds ratio, 12.1; 95% confidence interval, 2.01-72.79; p<0.01) as independent risk factors for high %WL.

Conclusion

Preventing high %WL by streamlining surgical procedures, minimizing postoperative complications, and implementing medical nutritional therapy (MNT) is imperative to improve the prognosis of patients with PDAC.

## Introduction

Malnutrition, a common problem among patients with pancreatic ductal adenocarcinoma (PDAC), negatively affects their quality of life (QOL) and treatment effectiveness. Despite the necessity of nutritional intervention as a routine procedure for patients with PDAC, no established recommendations or guidelines are available [1]. This underscores the pressing need to incorporate nutritional treatment into the multidisciplinary care process for these patients. Healthcare professionals, patients with cancer, and their families should be aware of the importance of nutritional status and medical nutritional therapy (MNT) in improving clinical outcomes and QOL for patients with PDAC [1]. PDAC is associated with a higher incidence of weight loss (WL) compared with other types of cancers [2], with approximately 80% of patients with PDAC experiencing weight loss upon diagnosis. More than one-third of these patients lose >10% of their body weight. Moreover, 70.3% of patients develop malnutrition during chemotherapy [3].

Weight loss is correlated with lower QOL and seems to be one of the most important symptoms of malnutrition. Several studies have investigated the association between perioperative WL and short- to long-term postoperative outcomes in esophageal [4], gastric [5-7], pancreatic [8-10], and oral [11] cancer patients.

Despite numerous studies [3-11] reporting on the correlation between pre- to postoperative weight loss or weight loss during chemotherapy and short- or long-term outcomes, few have focused on the parameter of early-stage WL during hospitalization immediately after surgical resection for patients with PDAC. In this study, we aimed to determine the association between WL during hospitalization after pancreatectomy and prognosis and identify risk factors influencing WL.

## Materials and methods

Patients

We included 68 patients diagnosed with PDAC who underwent radical pancreatectomy with regional lymphadenectomy at the Department of Surgery, Asahikawa Red Cross Hospital, between April 2017 and May 2023. The exclusion criteria for this study included patients diagnosed with intraductal papillary mucinous adenocarcinoma or pancreatic neuroendocrine tumor following postoperative pathological examination, along with those who underwent only exploratory laparotomy due to peritoneal dissemination and palliative surgeries, such as gastrointestinal bypass and choledochojejunostomy. All tumors were staged according to the eighth TNM classification system of the Union for International Cancer Control [12]. This study was approved by the Institutional Review Board of the Asahikawa Red Cross Hospital (approval number: 202332-2) and was conducted in compliance with the Declaration of Helsinki. The opt-out recruitment method was applied to all patients, allowing them to decline participation in the study.

Measurement of body weight

Patients diagnosed with PDAC and admitted to our department for radical surgery underwent weight assessments by medical staff upon admission and immediately prior to discharge. We used a digital weight scale (AD-6107NW, A&D, Japan) located in the inpatient ward. We defined the weight loss rate (%WL) as the percentage calculated by the formula: %WL = (weight at admission − weight before discharge)/weight at admission × 100. The association between %WL and the duration of postoperative hospitalization was also examined. Patients were classified into two groups based on %WL: a high %WL group, with a %WL of 10% or higher, and a low %WL group, with a %WL of 10% or lower. Subsequently, we analyzed their association with postoperative overall survival (OS) and postoperative recurrence-free survival (RFS).

Measurement of psoas muscle mass index and skeletal muscle mass index

Based on previous literature [13,14], the cross-sectional areas of the psoas and skeletal muscles at the level of the third lumbar vertebra were measured using the manual tracing method with preoperative CT images. Subsequently, we divided these areas by the square of the height to obtain the psoas muscle mass index (PMI) (cm^2^/m^2^) and the skeletal muscle mass index (SMI) (cm^2^/m^2^).

Nutritional assessment

To assess the preoperative nutritional status, objective nutritional indices, such as neutrophil-lymphocyte ratio (NLR), platelet-lymphocyte ratio (PLR), C-reactive protein-albumin ratio (CAR), modified Glasgow prognostic score (mGPS) [15], controlling nutritional status (CONUT) score [16], and Onodera prognostic nutritional index (PNI) [17], were obtained from blood biochemistry test data. The geriatric nutritional risk index (GNRI) was calculated using ideal body weight and albumin levels [18].

Statistical analysis

Differences between groups were analyzed using the Mann-Whitney U-test. Categorical variables were compared using Fisher’s exact test or the chi-square test. The correlation between the two groups was analyzed using Spearman’s correlation coefficients. OS was defined as the time from surgery to death from any cause. RFS was defined as the time from surgery to recurrence. The proportions of OS and RFS were calculated using the Kaplan-Meier method. Comparisons between the groups were performed using log-rank tests. Univariate and multivariate analyses were performed using a Cox proportional hazards model for prognostic analysis and a logistic regression model for predicting risk factors. Differences were considered statistically significant at p<0.05. Statistical analyses were performed using JMP Pro 17 (SAS Institute Inc., Cary, NC, USA).

## Results

Setting cut-off values using the receiver operating characteristic curve

The receiver operating characteristic (ROC) curve was used to set cut-off values for continuous variables, assuming a high risk of %WL immediately after surgery. Each continuous variable, along with its corresponding cut-off value, area under the curve (AUC), sensitivity, and specificity, is summarized in Table 1.

**Table 1:** Setting cut-off values for continuous variables. BMI: body mass index; SMI: skeletal muscle mass index; PMI: psoas muscle mass index; GNRI: geriatric nutritional risk index; NLR: neutrophil-lymphocyte ratio; PLR: platelet-lymphocyte ratio; CAR: C-reactive protein-albumin ratio; PNI: prognostic nutritional index.

Continuous variables	Cut-off value		AUC	Sensitivity	Specificity
Age		74	0.55	0.86	0.36
Operation time (min)		450	0.75	0.83	0.67
Blood loss (ml)		600	0.67	0.69	0.64
BMI (kg/m^2^)		22	0.52	0.55	0.56
SMI (cm^2^/m^2^)	Male	35.4	0.52	0.84	0.30
	Female	32.4	0.67	0.80	0.65
PMI (cm^2^/m^2^)	Male	4.7	0.59	0.79	0.48
	Female	3.6	0.59	0.70	0.53
GNRI		97	0.56	0.52	0.69
NLR		2.2	0.64	0.72	0.64
PLR		149	0.52	0.52	0.62
CAR		0.027	0.63	0.76	0.54
PNI		43	0.58	0.52	0.74

Relationship between %WL and postoperative hospital stay

**Figure 1:**
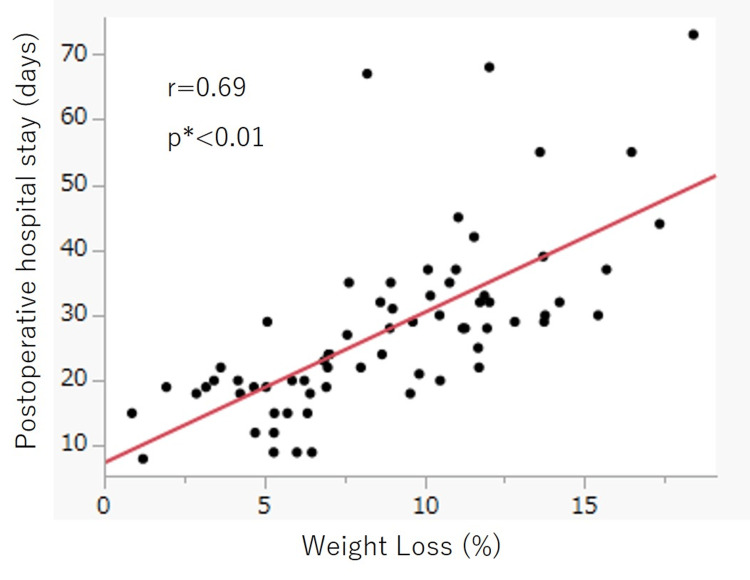
Relationship between %WL and postoperative hospital stay. %WL and postoperative hospital stay were positively correlated (r=0.69, p<0.01). WL: weight loss. *Spearman’s correlation coefficients test.

The median %WL for all patients was 8.8%. %WL and postoperative hospital stay were positively correlated (r=0.69, p<0.01; Figure 1).

Relationship between %WL and patients’ prognosis

Table 2 depicts a comparison of patient clinicopathological background characteristics between the high and low %WL groups.

**Table 2:** Clinicopathological background characteristics of the patients. ASA-PS: American Society of Anesthesiologists - Physical Status; Ph: pancreatic head; Pbt: pancreatic body or tail; PVR: portal vein resection; NAC: neoadjuvant chemotherapy. *Chi-square test.

Variables		Low %WL (n=39)	High %WL (n=29)	*p-value
Age	≧70	20	16	0.809
	＜70	19	13	
Gender	Male	23	19	0.622
	Female	16	10	
ASA-PS	1	13	13	0.634
	2	20	12	
	3	6	4	
Location	Ph	19	24	0.005
	Pbt	20	5	
PVR	With	5	11	0.022
	Without	34	18	
Transfusion	⁺	3	7	0.085
	−	36	22	
pT	0-2	7	5	1.000
	≧3	32	24	
pN	0	13	10	1.000
	1/2	26	19	
pM	0	37	29	0.504
	1	2	0	
NAC	⁺	15	13	0.627
	^-^​​​​​​	24	16	
Adjuvant	⁺	30	24	0.763
	^-^​​​​​​	9	5	
Complication	⁺	16	22	0.006
	^-^​​​​​​​​​​​​​	23	7	

No significant differences were found in age, sex, American Society of Anesthesiologists Physical Status (ASA-PS), presence of blood transfusion, pathological T, N, and M factors, or presence of preoperative and postoperative treatments between the two groups. The proportions of patients with pancreatic head cancer (p=0.005), portal vein resection (p=0.022), and postoperative complications (p=0.006) were significantly higher in the high %WL group than in the low %WL group.

**Figure 2:**
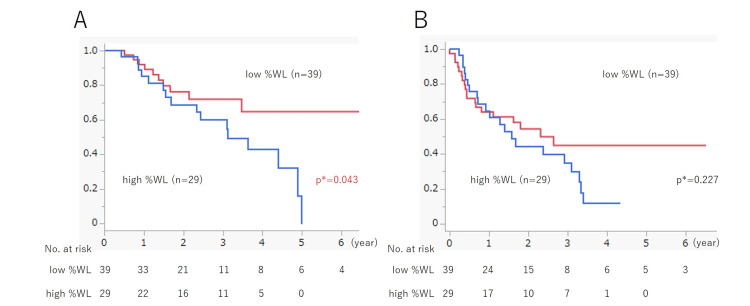
Relationship between %WL and patients’ prognosis. (A) Overall survival: the high %WL group had a significantly poorer prognosis than the low %WL group, with three- and five-year survival rates of 54.8% vs. 71.9% and 0% vs. 64.7%, respectively (p=0.043). (B) Relapse-free survival: there was a tendency for a poorer prognosis in the high %WL group, but the difference was not significant (p=0.227). WL: weight loss. *Log-rank test.

Survival analysis was performed using the Kaplan-Meier method. The median observation period for OS was 771.5 days. The high %WL group exhibited a significantly poorer prognosis than the low %WL group, with three- and five-year survival rates of 54.8% vs. 71.9% and 0% vs. 64.7%, respectively (p=0.043; Figure 2A). For RFS, the median observation period was 501 days. A tendency for a poorer prognosis in the high %WL group was observed, but the difference was not significant (p=0.227) (Figure 2B). Univariate analyses were performed for OS and RFS using the Cox proportional hazards model. Regarding OS, the hazard ratio for high %WL was 2.244 (95% confidence interval [CI], 1.006-5.006; p=0.048). Similarly, for RFS, the hazard ratio was 1.464 (95% CI, 0.785-2.729; p=0.230).

Analysis of risk factors influencing high %WL

We analyzed the risk factors influencing high %WL using logistic regression models. Univariate analysis identified several significant factors: pancreatic head cancer, operative time >450 min, blood loss >600 ml, portal vein resection, preoperative neutrophil-lymphocyte ratio >2.2, preoperative C-reactive protein-albumin ratio >0.027, and the presence of postoperative complications. In the multivariate analysis using only significant factors obtained from univariate analysis, operative time >450 min (odds ratio, 17.8; 95% CI, 1.01-312.42; p=0.049) and the presence of postoperative complications (odds ratio, 12.1; 95% CI, 2.01-72.79; p<0.01) were independent risk factors for high %WL (Table 3).

**Table 3:** Univariate and multivariate analyses for risk factors influencing %WL. OR: odds ratio; CI: confidence interval; Ph: pancreatic head; Pbt: pancreatic body or tail; ASA-PS: American Society of Anesthesiologists - Physical Status; PVR: portal vein resection; BMI: body mass index; PMI: psoas muscle mass index; SMI: skeletal muscle mass index; GNRI: geriatric nutritional risk index; NLR: neutrophil-lymphocyte ratio; PLR: platelet-lymphocyte ratio; CAR: C-reactive protein-albumin ratio; PNI: prognostic nutritional index; mGPS: modified Glasgow Prognostic Score; CONUT: controlling nutritional status. *Wald test.

N=68		Univariate analysis			Multivariate analysis		
Variables		OR	95%CI	*p-value	OR	95%CI	*p-value
Age (≧74/<74)		3.0	(0.94–9.57)	0.063			
Gender (Male/female)		1.3	(0.49–3.58)	0.583			
Location (Ph/Pbt)		5.1	(1.60–15.96)	0.006	0.7	(0.05–11.25)	0.824
ASA-PS (1/2,3)		1.6	(0.60–4.37)	0.336			
Operation time (≧450 min/<450 min)		9.6	(2.98–30.97)	<0.001	17.8	(1.01–312.42)	0.049
Blood loss (≧600 ml/<600 ml)		4.0	(1.43–11.04)	0.008	1.1	(0.19–6.66)	0.886
PVR (+/-)		4.2	(1.25–13.82)	0.020	4.7	(0.73–30.62)	0.103
Transfusion (+/−)		3.8	(0.89–16.32)	0.070			
Tumor size (≧2 cm/＜2 cm)		0.9	(0.30–2.92)	0.919			
Nodal metastasis (+/−)		1.0	(0.34–2.62)	0.921			
BMI (kg/m^2^) (≧22/＜22)		0.7	(0.28–1.89)	0.507			
SMI (cm^2^/m^2^)	Male (≧35.4/<35.4)	0.8	(0.30–2.35)	0.746			
	Female (≧32.4/＜32.4)						
PMI (cm2/m2)	Male (≧4.7/<4.7)	1.6	(0.60–4.39)	0.335			
	Female (≧3.6/＜3.6)						
GNRI (≧97/<97)		0.5	(0.18–1.29)	0.145			
NLR (≧2.2/<2.2)		0.3	(0.09–0.70)	0.008	0.2	(0.06–1.07)	0.062
PLR (≧149/<149)		0.7	(0.25–1.77)	0.419			
CAR (≧0.027/<0.027)		3.7	(1.27–10.56)	0.016	2.5	(0.53–11.98)	0.247
PNI (≧43/<43)		0.4	(0.13–1.05)	0.062			
mGPS (0/1.2)		0.4	(0.15–1.18)	0.102			
CONUT score (0.1/≧2)		0.8	(0.27–2.18)	0.612			
Complication (+/−)		4.5	(1.56–18.08)	0.005	12.1	(2.01–72.79)	0.007

## Discussion

In this study, we focused on a novel parameter, %WL, monitored during the immediate post-radical surgery hospitalization of patients with PDAC. We observed that patients in the high %WL group, who experienced a weight loss of 10% or more at admission, exhibited a significantly poorer prognosis for OS than those in the low %WL group, with a hazard ratio of 2.244 (95% CI, 1.006-5.006; p=0.048) as per the univariate analysis. Multivariate analysis using logistic regression models demonstrated that an operation time of 450 min or longer (odds ratio, 17.8; 95% CI, 1.01-312.42; p=0.049) and the development of postoperative complications (odds ratio, 12.1; 95% CI, 2.01-72.79; p<0.01) were independent risk factors affecting high %WL during hospitalization. Actually, the cutoff value of 450 min of operation time showed the highest AUC (0.75) for the ROC curve, with a sensitivity of 83% and a specificity of 67%. These results suggest that prolonged surgery and the development of postoperative complications in patients with PDAC may contribute to a high %WL during the postoperative period and negatively affect long-term prognosis.

Previous studies on patients with gastric cancer reported that a weight loss of 15% or more within the first month after surgery affects the continuity of S-1 adjuvant chemotherapy and results in a significantly poorer five-year survival rate [5,6]. Similarly, research involving patients with pancreatic cancer shows that a weight loss of 10% or more during hospitalization results in poor RFS by lowering the relative dose intensity of postoperative adjuvant chemotherapy and affecting its continuity [9]. Why does weight loss affect postoperative adjuvant chemotherapy? A possible reason is the impact of decreased lean body mass on chemotherapy-induced toxicity [5]. Prado et al. reported that severe skeletal muscle depletion is a significant predictor of 5-fluorouracil toxicity in metastatic breast cancer [19]. Another possible reason is the decrease in physiological function with WL. Even modest weight loss, such as 5% of body weight, can alter various physiological parameters, including immune response, lung and cardiac function tests, and autonomic autoregulation [20]. Notably, although previous reports have addressed this issue, our study found no significant difference in the percentage of patients receiving postoperative adjuvant chemotherapy between those with high and those with low %WL. This suggests that postoperative adjuvant chemotherapy did not impact this study's OS or RFS results.

A high %WL may have affected postoperative OS due to several reasons. Pancreatic surgery is associated with significant postoperative morbidity, mortality, and prolonged hospital stay [21]. Although technological advances in surgical techniques and perioperative management have greatly improved mortality rates after pancreatic resection, postoperative morbidity remains a significant critical concern [22]. Pancreatic resection has been identified as one of the most complex surgical procedures owing to its extended resection, resulting in metabolic stress and a comparatively high rate of complications. This type of surgery strongly modifies metabolic activity and nutritional conditions by triggering inflammation, stress hormones, and cytokines [23]. Notably, inflammation and its catabolic effects play important roles in the development of cachexia. The catabolic effects resulting from inflammation influence weight loss and the development of sarcopenia and mediate several cytokines released by the cancer itself and the patient’s system. In PDAC cachexia, interleukin (IL)-1, IL-6, IL-8, and tumor necrosis factor α were identified as the most influential factors [24].

In this study, the high %WL group exhibited significantly more cases of pancreatic head cancer (necessitating pancreatoduodenectomy), portal vein resection, and postoperative complications than the low %WL group, which may be important factors to consider in relation to prognosis. Table 4 provides a breakdown of postoperative complications.

**Table 4:** A breakdown of postoperative complications in patients with pancreatic ductal adenocarcinoma. CD: Clavien-Dindo classification; POPF: postoperative pancreatic fistula; SSI: surgical site infection; DGE: delayed gastric empty.

Complications		N (%)
All		38 (55.9)
CD ≧ 3		26 (38.2)
POPF	BL	5 (7.3)
	BL	18 (26.5)
	C	1 (1.5)
Anastomosis trouble	Leakage	4 (5.9)
	Hemorrhage	3 (4.4)
	Stenosis	1 (1.5)
SSI	Incisional	1 (1.5)
	Organ/space	3 (4.4)
Chylous ascites		2 (2.9)
DGE		1 (1.5)
Cholangitis		1 (1.5)

Complications occurred in 38 patients (55.9%), with 26 cases (38.2%) classified as Clavien-Dindo grade [25] ≧3. Pancreatic fistula was identified as the most common complication, occurring in 24 patients (35.3%), graded according to the International Study Group on Pancreatic Surgery as follows [26]: BL, 5 patients (7.3%); B, 18 patients (26.5%); and C, 1 patient (1.5%). Anastomotic problems occurred in eight patients (11.8%), with four patients (5.9%) experiencing leakage, three patients (4.4%) encountering bleeding, and one patient (1.5%) developing stenosis. Surgical site infections (SSI) occurred in four patients (5.9%), including one (1.5%) incisional wound and three (4.4%) body cavities/organs. Chylous ascites occurred in two patients (2.9%). Postoperative cholangitis occurred in one patient (1.5%). No morbidity or mortality were observed. Generally, pancreatoduodenectomy is a more complex procedure than distal pancreatectomy, and its operative time is longer because of the reconstruction involved. The development and progression of cancer-related malnutrition (CRM) are associated with reduced oral nutritional intake and/or increased catabolism [27]. Based on the differences in clinicopathological background characteristics between the high and low %WL groups in this study, it can be inferred that patients with pancreatic head cancer who underwent concomitant portal vein resection had higher complication rates, were more invasive, released more stress hormones and inflammatory cytokines, and were more hypercatabolic, resulting in severe weight loss. Moreover, CRM may affect long-term outcomes.

The prevention of postoperative weight loss is important for improving the prognosis of patients with PDAC. In this study, multivariate analysis identified operation time >450 min and the development of postoperative complications as independent risk factors influencing postoperative high %WL. Addressing these factors involves two strategies: reducing operation time and minimizing the occurrence of complications after surgery. Understanding anatomy through preoperative imaging data and detailed surgical simulations covering possible scenarios is important. Standardization of surgical procedures contributes to achieving consistent surgical outcomes. Nontechnical skills among surgical team members are also considered important. Nevertheless, pancreatectomy, being a complex procedure, presents challenges in minimizing the occurrence of postoperative complications. Therefore, nutritional assessment and MNT are important in mitigating postoperative complications and preventing excessive weight loss, particularly in cases of prolonged hospital stay. The early detection of signs of malnutrition is crucial not only during diagnosis but throughout all stages of treatment [1]. Nutritional counseling should precede the initiation of treatment for PDAC and continue throughout and after treatment [1]. Early dietary counseling among patients with PDAC not only enhances their nutritional status but also improves survival rates [28]. For patients facing challenges in achieving 100% of their macro- and micronutrient requirements through diet alone, the use of oral nutritional supplements may be an effective strategy after nutritional counseling, providing essential support [29]. A systematic review of patients with gastrointestinal (gastric, esophageal, and pancreatic) cancer undergoing surgery revealed limited evidence supporting the effectiveness of oral nutritional supplements in promoting weight gain and increased energy intake both before and after surgery [30]. During pancreatic surgery, early prevention of malnutrition and provision of proper perioperative MNT for CRM are required to improve tolerance to antineoplastic therapy and clinical outcomes [31]. For proper MNT, a multidisciplinary approach may be necessary for patients with CRM after pancreatic surgery.

This study had several limitations. First, it was a single-institute retrospective cohort study with a small sample size. Second, the timing of body weight measurements and the examiners were inconsistent, possibly resulting in measurement bias. Third, the duration of preoperative or postoperative chemotherapy, regimens used, and dose intensity varied between patients, potentially affecting long-term outcomes. Fourth, the assessment of postoperative complications and nutritional management, such as parenteral or enteral nutrition therapy, varied depending on the attending physician, potentially influencing WL during hospitalization. A large number of multi-institutional prospective studies are required to confirm our findings.

## Conclusions

In conclusion, we focused on a novel parameter, WL, during hospitalization after radical resection for PDAC and demonstrated that a high %WL was associated with a worse prognosis. Prolonged surgery and postoperative complications were independent risk factors for high %WL. To further improve the prognosis of patients with PDAC, surgical procedures must be performed in a shorter time with fewer complications. Early nutritional assessment and intervention are important for preventing WL.

## References

[1] Mękal D, Sobocki J, Badowska-Kozakiewicz A (2023). Evaluation of nutritional status and the impact of nutritional treatment in patients with pancreatic cancer. Cancers (Basel).

[2] Arends J, Baracos V, Bertz H (2017). ESPEN expert group recommendations for action against cancer-related malnutrition. Clin Nutr.

[3] Bundred J, Kamarajah SK, Roberts KJ (2019). Body composition assessment and sarcopenia in patients with pancreatic cancer: a systematic review and meta-analysis. HPB (Oxford).

[4] Hirano Y, Konishi T, Kaneko H (2023). Weight loss during neoadjuvant therapy and short-term outcomes after esophagectomy: a retrospective cohort study. Int J Surg.

[5] Aoyama T, Yoshikawa T, Shirai J (2013). Body weight loss after surgery is an independent risk factor for continuation of S-1 adjuvant chemotherapy for gastric cancer. Ann Surg Oncol.

[6] Aoyama T, Sato T, Maezawa Y (2017). Postoperative weight loss leads to poor survival through poor S-1 efficacy in patients with stage II/III gastric cancer. Int J Clin Oncol.

[7] Segami K, Aoyama T, Kano K (2018). Risk factors for severe weight loss at 1 month after gastrectomy for gastric cancer. Asian J Surg.

[8] Hashimoto D, Chikamoto A, Ohmuraya M (2015). Impact of postoperative weight loss on survival after resection for pancreatic cancer. JPEN J Parenter Enteral Nutr.

[9] Morita Y, Sakaguchi T, Kitajima R (2019). Body weight loss after surgery affects the continuity of adjuvant chemotherapy for pancreatic cancer. BMC Cancer.

[10] Nishikawa M, Yamamoto J, Einama T (2022). Preoperative rapid weight loss as a prognostic predictor after surgical resection for pancreatic cancer. Pancreas.

[11] Mohammed RA, Ahmed SK (2024). Nutritional support for oral cancer patients: what every nurses 
should know?. Oral Oncol Rep.

[12] Brierley JD, Gospodarowicz MK, Wittekind C (2017). TNM Classification of Malignant Tumours, 8th edition. Hoboken: Wiley.

[13] Okumura S, Kaido T, Hamaguchi Y (2015). Impact of preoperative quality as well as quantity of skeletal muscle on survival after resection of pancreatic cancer. Surgery.

[14] Prado CM, Lieffers JR, McCargar LJ (2008). Prevalence and clinical implications of sarcopenic obesity in patients with solid tumours of the respiratory and gastrointestinal tracts: a population-based study. Lancet Oncol.

[15] Wang Y, Chen L, Wu Y, Li P, Che G (2020). The prognostic value of modified Glasgow prognostic score in patients with esophageal squamous cell cancer: a Meta-analysis. Nutr Cancer.

[16] Ignacio de Ulíbarri J, González-Madroño A, de Villar NG (2005). CONUT: a tool for controlling nutritional status. First validation in a hospital population. Nutr Hosp.

[17] Onodera T, Goseki N, Kosaki G (1984). Prognostic nutritional index in gastrointestinal surgery of malnourished cancer patients. Nihon Geka Gakkai Zasshi.

[18] Bouillanne O, Morineau G, Dupont C (2005). Geriatric Nutritional Risk Index: a new index for evaluating at-risk elderly medical patients. Am J Clin Nutr.

[19] Prado CM, Baracos VE, McCargar LJ (2009). Sarcopenia as a determinant of chemotherapy toxicity and time to tumor progression in metastatic breast cancer patients receiving capecitabine treatment. Clin Cancer Res.

[20] Lennard-Jones JE A positive approach to nutrition as treatment. Report of a Working Party.

[21] Nimptsch U, Krautz C, Weber GF, Mansky T, Grützmann R (2016). Nationwide in-hospital mortality following pancreatic surgery in Germany is higher than anticipated. Ann Surg.

[22] Greenblatt DY, Kelly KJ, Rajamanickam V (2011). Preoperative factors predict perioperative morbidity and mortality after pancreaticoduodenectomy. Ann Surg Oncol.

[23] Gianotti L, Besselink MG, Sandini M (2018). Nutritional support and therapy in pancreatic surgery: a position paper of the International Study Group on Pancreatic Surgery (ISGPS). Surgery.

[24] Rovesti G, Valoriani F, Rimini M (2021). Clinical implications of malnutrition in the management of patients with pancreatic cancer: introducing the concept of the Nutritional Oncology Board. Nutrients.

[25] Clavien PA, Barkun J, de Oliveira ML (2009). The Clavien-Dindo classification of surgical complications: five-year experience. Ann Surg.

[26] Bassi C, Marchegiani G, Dervenis C (2024). Corrigendum to "The 2016 update of the International Study Group (ISGPF) definition and grading of postoperative pancreatic fistula: eleven years after." Surgery 2017. Mar; 161 (3):584-591. Epub Dec 28, 2016. Surgery.

[27] Fearon K, Strasser F, Anker SD (2011). Definition and classification of cancer cachexia: an international consensus. Lancet Oncol.

[28] Martin D, Joliat GR, Halkic N, Demartines N, Schäfer M (2020). Perioperative nutritional management of patients undergoing pancreatoduodenectomy: an international survey among surgeons. HPB (Oxford).

[29] Carrato A, Cerezo L, Feliu J (2022). Clinical nutrition as part of the treatment pathway of pancreatic cancer patients: an expert consensus. Clin Transl Oncol.

[30] Cintoni M, Grassi F, Palombaro M (2023). Nutritional interventions during chemotherapy for pancreatic cancer: a systematic review of prospective studies. Nutrients.

[31] Menozzi R, Valoriani F, Ballarin R (2023). Impact of nutritional status on postoperative outcomes in cancer patients following elective pancreatic surgery. Nutrients.

